# Novel Rhodanine Derivative, 5-[4-(4-Fluorophenoxy) phenyl]methylene-3-{4-[3-(4-methylpiperazin-1-yl) propoxy]phenyl}-2-thioxo-4-thiazolidinone dihydrochloride, Induces Apoptosis via Mitochondria Dysfunction and Endoplasmic Reticulum Stress in Human Colon Cancer Cells

**DOI:** 10.3390/molecules23112895

**Published:** 2018-11-06

**Authors:** Hye-Uk Jung, Jeong-Hun Lee, Kyung-Sook Chung, Joo Young Hong, Jung-Hye Choi, Soo-Dong Kim, Eun Joo Roh, Kye Jung Shin, Kyung-Tae Lee

**Affiliations:** 1 Department of Pharmaceutical Biochemistry, College of Pharmacy, Kyung Hee University, Seoul 130-701, Korea; 1109jhuk@naver.com (H.-U.J.); ztztzt08@hanmail.net (J.-H.L.); adella76@hanmail.net (K.-S.C.); hjisuk1206@naver.com (J.Y.H.); 2 Department of Life and Nanopharmaceutical Science, Graduate School, Kyung Hee University, Seoul 130-701, Korea; jchoi@khu.ac.kr; 3 Department of Oriental Pharmaceutical Science, College of Pharmacy, Kyung Hee University, Seoul 130-701, Korea; 4 Department of Urology, College of Medicine, Dong-A University, Pusan 602-715, Korea; rose1508@naver.com; 5 Life/Health Division, Korea Institute of Science and Technology, 5Wolsong-gil, Seongbuk-gu, Seoul 136-791, Korea; heesu3620@hanmail.net; 6 Integrated Research Institute of Pharmaceutical Sciences, College of Pharmacy, The Catholic University of Korea, 43 Jibong-ro, Wonmi-gu, Bucheon, Gyeonggi-do 420-743, Korea

**Keywords:** rhodanine derivative, apoptosis, colon cancer, ER stress, calcium, mitochondria

## Abstract

We previously reported that 5-[4-(4-fluorophenoxy) phenyl] methylene-3-{4-[3-(4-methylpiperazin-1-yl)propoxy]phenyl}-2-thioxo-4-thiazolidinone dihydrochloride (KSK05104) has potent, selective and metabolically stable IKKβ inhibitory activities. However, the apoptosis-inducing of KSK05104 and its underlying mechanism have not yet been elucidated in human colon cancer cells. We show that KSK05104 triggered apoptosis, as indicated by externalization of Annexin V-targeted phosphatidylserine residues in HT-29 and HCT-116 cells. KSK05104 induced the activation of caspase-8, -9, and -3, and the cleavage of poly (ADP ribose) polymerase-1 (PARP-1). KSK05104-induced apoptosis was significantly suppressed by pretreatment with z-VAD-fmk (a broad caspase inhibitor). KSK05104 also induced release of cytochrome *c* (Cyt *c*), apoptosis inducing factor (AIF), and endonuclease G (Endo G) by damaging mitochondria, resulting in caspase-dependent and -independent apoptotic cell death. KSK05104 triggered endoplasmic reticulum (ER) stress and changed the intracellular calcium level ([Ca^2+^]_i_). Interestingly, treatment with KSK05104 activated not only ER stress marker proteins including inositol-requiring enzyme 1-alpha (IRE-1α) and protein kinase RNA-like endoplasmic reticulum kinase (PERK), but also μ-calpain, and caspase-12 in a time-dependent manner. KSK05104-induced apoptosis substantially decreased in the presence of BAPTA/AM (an intracellular calcium chelator). Taken together, these results suggest that mitochondrial dysfunction and ER stress contribute to KSK05104-induced apoptosis in human colon cancer cells.

## 1. Introduction

The colon and rectum both belong to the digestive system of humans, and cancers affecting either of these organs are known as colorectal cancer (CRC). Globally, CRC is a leading cause of cancer-related deaths, with over 66,000 annual deaths reported [1]. For a long time, various formulations containing 5-Fluorouracil (5-FU) have been administered through different routes as standard treatment for CRC. Therapy with irinotecan and oxaliplatin has also improved therapeutic outcomes of this disease [2]. However, such approaches are of limited value for advanced or recurrent CRC.

Apoptosis, the primary form of programmed cell death, is essential to numerous biological events [3]. The intrinsic apoptosis pathway begins with mitochondrial outer membrane permeabilization (MOMP), for which mitochondrial intra-membranous oligomerization of Bax/Bak is important [4]. Bax/Bak comprise an essential gateway for mitochondrial apoptosis in that their combined deletion prevents the release of cytochrome *c* (Cyt *c*) and results in resistance to all death stimuli that activate the intrinsic apoptosis pathway [5]. Bax is located mainly in the cytosol, with a minor pool loosely attached to mitochondria, and following certain apoptotic stimuli, BH3-only proteins can induce structural changes in the Bax structure and expose its N-terminus that inserts and subsequently translocates into the mitochondrial outer membrane (MOM) [6]. In contrast with Bax, Bak is constitutively inserted in the MOM, where it is bound to and inhibited by VDAC2, Mcl-1, and Bcl-xL [7]. Similar as Bax, Bak oligomerization requires BH3-only proteins to induce mitochondrial membrane potential (*ΔΨ_m_*) [8], and after a loss in membrane permeability, apoptotic proteins including Cyt *c*, apoptosis inducing factor (AIF), endonuclease G (Endo G), and second mitochondria-derived activator of caspase/direct inhibitor of apoptosis protein (IAP)-binding protein with low pI (Smac/DIABLO) are released from the mitochondrial inter-membrane space into the cytosol. Cyt *c* and Smac/DIABLO mainly mediate the caspase-dependent apoptotic pathway by activating caspase-9 and -3 [9,10], whereas AIF and Endo G mediate the caspase-independent apoptotic pathway [11]. AIF is central in caspase-independent apoptosis [12]. After apoptotic mulation, AIF is cleaved to be a soluble 57 kDa fragment that is released from mitochondria and is directly translocated to the nucleus, where it promotes chromatin condensation and large-scale DNA fragmentation [12].

The endoplasmic reticulum (ER) is another critical intracellular organelle for apoptosis [13], and it is also essential to ensure correct folding/assembly, glycosylation, and sorting of proteins in the secretory system [14]. The ER is also involved in intracellular calcium homeostasis [15]. Apoptotic cell death ensues when the ER stress is too extensive or prolonged [16], and important mediators of ER stress-associated apoptosis include the activation of procaspase-12, as well as increased expression of pro-apoptotic transcription factor GADD153/CHOP [17].

**Figure 1:**
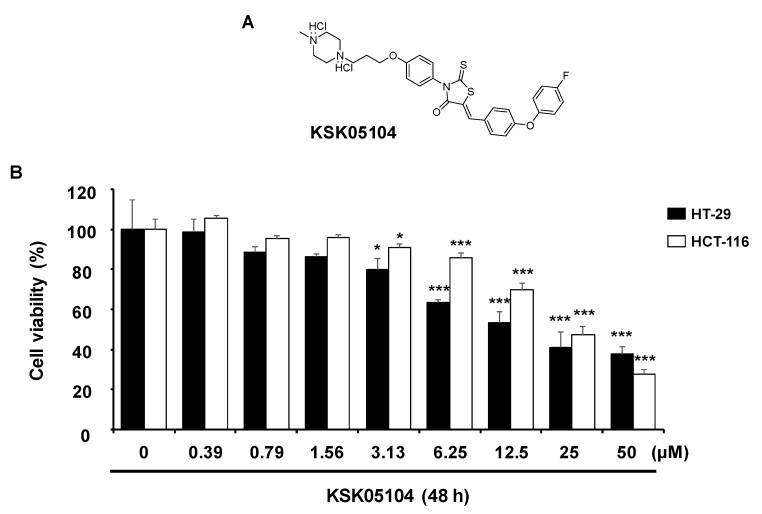
Effect of KSK05104 on the growth inhibition in colon cancer cells. (**A**) Chemical structure of KSK05104; (**B**) Cell viability of KSK05104 in HT-29 and HCT-116 cells. Cells were treated with KSK05104 for the indicated times. Data are the means ± S.D. of the results from three independent experiments. * *p* < 0.05, *** *p* < 0.001 vs. non-treated control group.

As a program for searching compounds for showing an inhibitory activity on IKKβ in our in-house library by high-throughput screening (HTS), a hit compound having a rhodanine ring as a core structure is employed. Thus, rhodanine-based compounds will probably continue to be pivotal and important as scaffolds for drug discovery [18]. Rhodanine derivatives exhibited various biological characteristics such as anti-convulsant, anti-bacterial, anti-viral, and anti-diabetic activities [19]. One of their anti-viral functions involves inhibiting hepatitis C virus (HCV) protease [20] and also various enzymes including bacterial β-lactamase and muramyl ligases and uridine diphospho-*N*-acetylmuramate/l-alanine ligase [21]. In our continued efforts to discover novel compounds with potent anti-tumor effects, we synthesized the rhodanine derivatives as a stable IKKβ inhibitor [22]. Among the rhodanine derivatives, 5-[4-(4-fluorophenoxy) phenyl]methylene-3-{4-[3-(4-methylpiperazin-1-yl)propoxy]phenyl}-2-thioxo-4-thiazolidinone dihydrochloride (KSK05104; Figure 1A) showed excellent selectivity IKKβ over other kinases and had a high potential as drug candidate in the development of therapeutics for the treatment of NF-κB associated immune disease such as cancer. An association of colorectal cancer development and activation of NF-κB has been well demonstrated [23,24]. NF-κB activity was increased in the colon cancer cell lines and human tumor samples as well as nucleic of stromal macrophages in sporadic adenomatous polyps. Thus, inactivation of NF-κB in colon cancer cells by IKK inhibitors was demonstrated to blunt the ability of the cancer cells to grow by the antiapoptotic function of NF-κB. Attenuation of the IKK/NF-κB signaling pathway led to inhibition of the target gene that can interfere with the apoptotic process [25]. Hence, in this study, we evaluated the cytotoxicity and molecular mechanisms involving the pro-apoptotic activities of KSK05104 in HT-29 human colon adenocarcinoma cells.

## 2. Results

### 2.1. KSK05104 Induces the Growth Inhibition in HT-29 Cells

To determine the cytotoxicity of KSK05104, dose-response effects were examined using an 3-(4,5-dimethylthiazol-2-yl)-2,5-diphenyltetrazolium bromide (MTT) assay in human colon cancer cells. As shown in Figure 1B, KSK05104 displayed a cytotoxic effect on HT-29 and HCT-116 cells (IC_50_ = 15.93 ± 2.41 μM and 31.96 ± 1.56 μM, respectively). On the other hand, KSK05104 showed IC_50_ values of 88.79 ± 2.91 μM (CCD-18Co), 90.06 ± 3.55 μM (L132) and 45.8 ± 3.69 μM (IOSE-80PC) in normal cell lines, indicating that KSK05104 has less cytotoxic effect on normal cells, at least in colon fibroblast and lung epithelial cells, compared with colon cancer cells (Table 1).

### 2.2. KSK05104 Induces Apoptosis in HT-29 Cells

**Figure 2:**
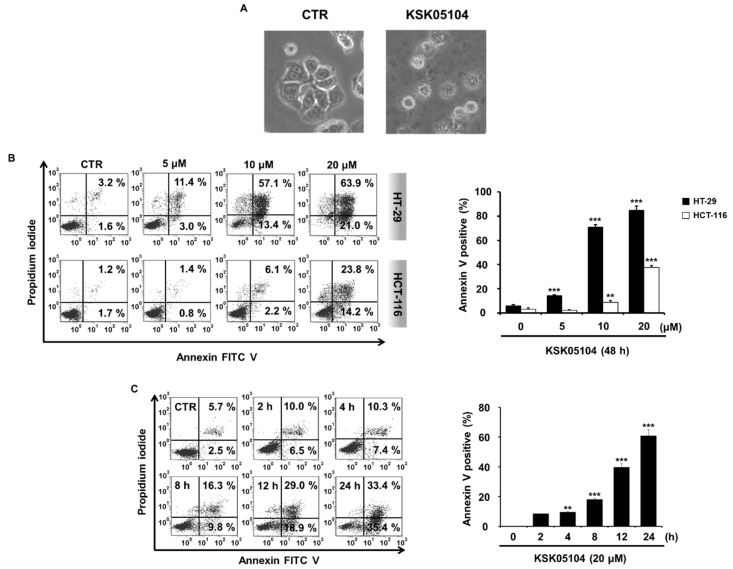
Effect of KSK05104 on apoptosis induction in colon cancer cells. (**A**) Representative image showed the change of cell morphology in 20 µM KSK05104-treated HT-29 cells for 24 h; (**B**) HT-29 and HCT-116 cells were treated with various concentration of KSK05104 for 48 h; and (**C**) HT-29 cells were treated with 20 µM KSK05104 for the indicated times. Cells were co-stained with propidium iodide (PI) and fluorescein-5-isothiocyanate (FITC)-conjugated Annexin V, and the translocation of phosphatidylserine (PS) was detected via flow cytometry after KSK05104 treatment. Data are the means ± S.D. of the results from three independent experiments. ** *p* < 0.01, *** *p* < 0.001 vs. non-treated control group.

During apoptosis, cells display typical apoptotic morphology, with cell retraction and dynamic plasma membrane blebbing and show externalization of phosphatidylserine (PS). To more precisely determine the cytotoxic effect of KSK05104 involved apoptosis, we observed cell morphology with a microscope and we examined the translocation of PS using Annexin V and PI double staining. As shown in Figure 2A, KSK05104-treated cells collapse into small intact fragments termed apoptotic bodies and plasma membrane blebbing was observed. In addition, treatment of 20 µM KSK05104 resulted in a concentration-dependent increase of externalized PS indicating the early and late stages of apoptosis in HT-29 and HCT-116 cells (Figure 2B). Moreover, a time-dependent increase in PS was also measured in KSK05104-treated HT-29 cells (Figure 2C).

### 2.3. KSK05104-Induced Apoptosis Requires Caspase Activation in HT-29 Cells

**Figure 3:**
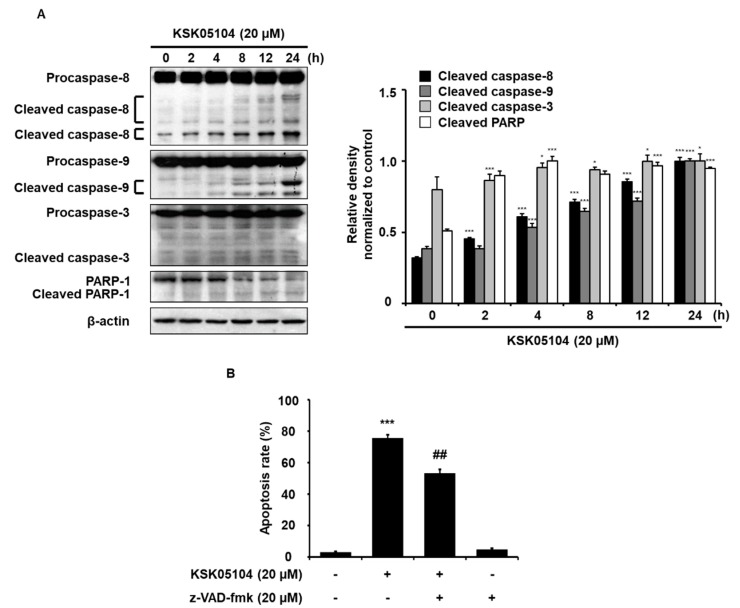
Effect of KSK05104 on the activation of caspases in HT-29 cells. (**A**) HT-29 cells were treated with 20 µM KSK05104 for the indicated times to examine the expression of caspase-8, -9, -3 and PARP-1 via Western blot analysis. Ratio of relative density was determined by a densitometric analysis program (Bio-rad Quantity One^®^ Software) normalized to internal control; (**B**) Cells were pretreated with 20 µM z-VAD-fmk for 1 h and then treated with or without 20 µM KSK05104 for 24 h. Cells were co-stained with PI and FITC-conjugated Annexin V, and the translocation of PS was detected by flow cytometry after treatment with KSK05104. Data presented are the means ± S.D. of results from three independent experiments. * *p* < 0.05, *** *p* < 0.001 vs. non-treated control group, *^##^ p* < 0.01 vs. KSK05104-treated control group.

To investigate the cell signaling involved in KSK05104-induced apoptosis, we examined whether treatment with KSK05104 leads to caspase activation in HT-29 cells. KSK05104 significantly and time-dependently increased the activation of caspase-8, -9, and -3, and the cleavage of PARP-1, an endogenous substrate of caspase-3 (Figure 3A). To further confirm the involvement of caspases in KSK05104-induced apoptosis, HT-29 cells were treated with 20 µM z-VAD-fmk, a broad caspase inhibitor. z-VAD-fmk partially suppressed KSK05104-induced apoptosis (Figure 3B).

### 2.4. KSK05104 Modulates the Expression of Bcl-2 Family Proteins and Reduces ΔΨ_m_ in HT-29 Cells

**Figure 4:**
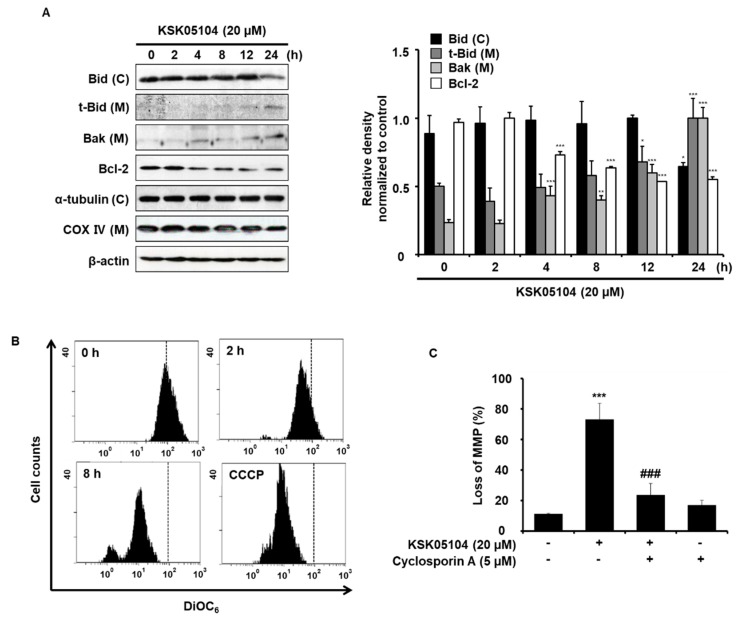
Effect of KSK05104 on the expression of Bcl-2 family proteins and mitochondrial membrane potential (*ΔΨ_m_*) in HT-29 cells. (**A**) Cells were treated with 20 µM KSK05104 for 24 h and were then harvested. Mitochondrial (M) and cytosolic (C) fractions and total cell lysates were prepared. The expression of Bid, Bak and Bcl-2 was examined via Western blot analysis. α-Tubulin, COX IV, and β-actin were used as internal controls. The ratio of relative density was determined by a densitometric analysis program (Bio-rad Quantity One^®^ Software) normalized to internal control; (**B**) Cells were treated with 20 µM KSK05104 for 24 h, stained with 40 nM DiOC_6_, and analyzed via flow cytometry. Carbonyl cyanide m-chlorophenyl hydrazine (CCCP) (100 µM) was used as positive control; (**C**) Cells pretreated with 5 μM CsA for 30 min and then treated with 20 μM KSK05104 for 8 h, stained with DiOC_6_, and analyzed via fluorescence-activated cell sorting (FACS). Data are the means ± S.D. of results from three independent experiments. * *p* < 0.05, ** *p* < 0.01, *** *p* < 0.001 vs. non-treated control group; ^###^ *p* < 0.001 vs. KSK05104-treated control group.

The Bcl-2 family of proteins regulates the apoptosis that occurs via the mitochondrial pathway by maintaining a balance between pro- and anti-apoptotic members [26]. Thus, we examined whether KSK05104 has any effects on the levels of Bcl-2 family proteins in HT-29 cells. KSK05104 significantly decreased the cytosolic levels of Bid in 24 h, but increased the mitochondrial levels of t-Bid and Bak. In addition, KSK05104 treatment reduced the levels of Bcl-2 protein expression in HT-29 cells. (Figure 4A). In this regard, we examined the effect of KSK05104 on the *ΔΨ_m_*, as measured by flow cytometry in HT-29 cells using DiOC_6_. Similar to carbonyl cyanide m-chlorophenyl hydrazine (CCCP), which was used as positive control, KSK05104 treatment significantly and time-dependently decreased *ΔΨ_m_* (Figure 4B). These findings indicate that KSK05104 modulates the levels of the Bid, Bak, and Bcl-2 proteins, resulting in a loss in *ΔΨ_m_*. To confirm whether KSK05104 decreases *ΔΨ_m_*, we used Cyclosorin A (CsA), an inhibitor of mitochondrial permeability transition. Pretreatment with CsA significantly attenuated KSK05104-induced loss in *ΔΨ_m_* in HT-29 cells (Figure 4C).

### 2.5. KSK05104 Induces the Translocation of Mitochondrial Apoptogenic Factors in HT-29 Cells

**Figure 5:**
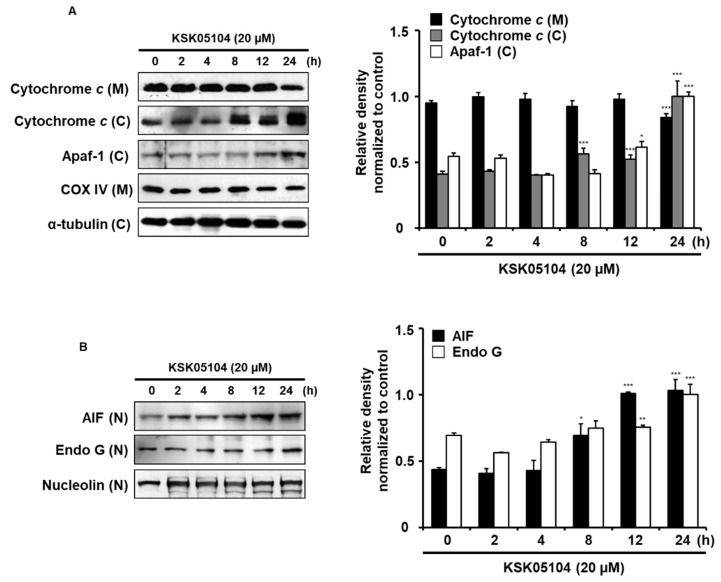
Effect of KSK05104 on the translocation of mitochondrial apoptotic factors in HT-29 cells. (**A**,**B**) The cells were treated with 20 µM KSK05104 for 24 h and then harvested. Mitochondrial (M), cytosolic (C), and nuclear (N) fractions and total cell lysates were prepared. Expression of Cyt *c*, Apaf-1, apoptosis inducing factor (AIF), and Endo G were examined via Western blot analysis. COX IV, α-tubulin and nucleolin were used as internal controls and data are presented by results from three independent experiments. Ratio of relative density was determined by a densitometric analysis program (Bio-rad Quantity One^®^ Software) normalized to internal control. * *p* < 0.05, ** *p* < 0.01, *** *p* < 0.001 vs. non-treated control group.

A disruption in *ΔΨ_m_* is generally linked to the translocation of Cyt *c* and AIF into cytosol located between the outer and inner mitochondrial membranes [12]. Cytosolic Cyt *c* activates caspase-9 by binding to apoptotic protease activating factor 1 (Apaf-1), which leads to caspase-9 activation and subsequent activations of downstream executioner caspases. The cytosolic Cyt *c* and Apaf-1 level was elevated by KSK05104 in HT-29 cells (Figure 5A), which provided a clear indication that KSK05104 induced caspase-dependent apoptosis in HT-29 cells. However, the observation that z-VAD-fmk did not completely inhibit the apoptotic effect of KSK05104 (Figure 3B) indicated an involvement of a caspase-independent apoptotic pathway. AIF induces caspase-independent apoptosis by directly inducing DNA fragmentation [27]. To determine whether AIF is involved in KSK05104-induced caspase-independent apoptosis, we conducted a Western blot analysis to examine the effect that KSK05104 had on AIF and Endo G nuclear translocation. KSK05104 treatment increased the nuclear translocation of AIF and Endo G in a time-dependent manner (Figure 5B). These results suggest that KSK05104-induced apoptosis is involved in both caspase-dependent and -independent pathways through mitochondrial-mediated apoptosis.

### 2.6. KSK05104 Increases Intracellular Calcium Level and ER Stress-Mediated Apoptosis in HT-29 Cells

**Figure 6:**
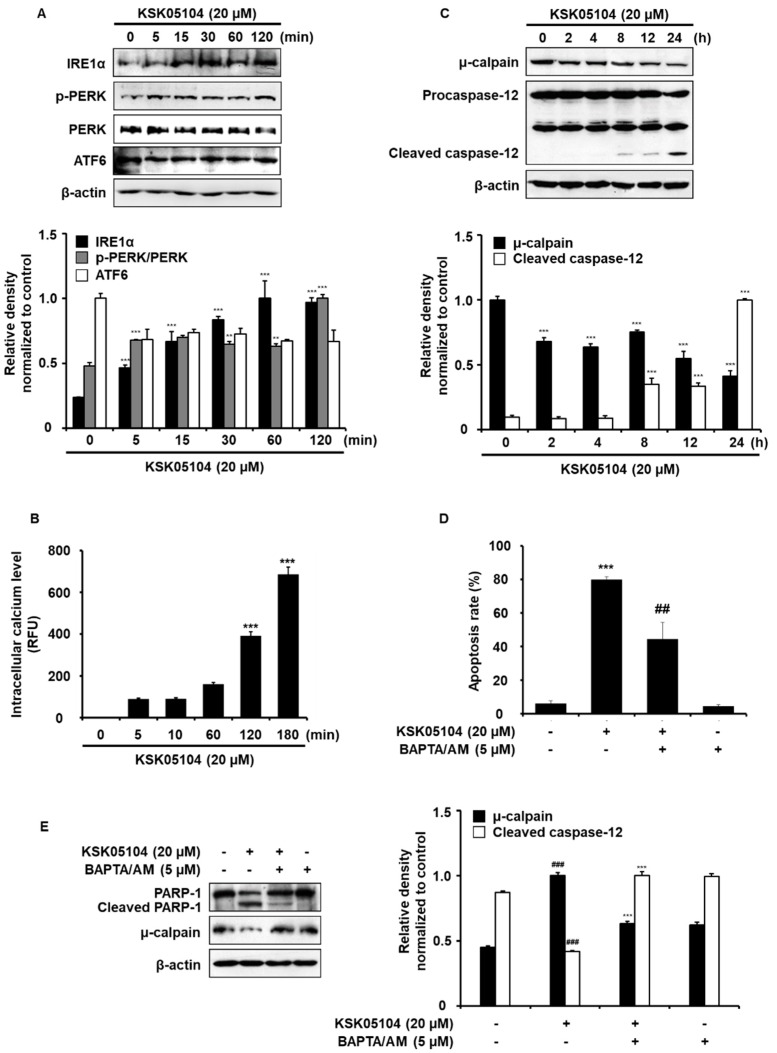
Effect of KSK05104 on endoplasmic reticulum (ER) stress and Ca^2+^-mediated apoptosis in HT-29 cells. (**A**) Cells treated with 20 µM KSK05104 for 2 h. Western blot analysis was conducted using antibodies against IRE-1α, p-PERK, PERK, and ATF6. β-actin was used as internal control. Ratio of relative density was determined by a densitometric analysis program (Bio-rad Quantity One^®^ Software) normalized to internal control; (**B**) Cells were treated with 20 µM KSK05104 for the indicated times, and the level of the [Ca^2+^]_i_ was determined using a Fluo-4/AM fluorescence kit. The stained cells were analyzed using fluorescence; (**C**) Cells treated with 20 µM KSK05104 for 24 h. Western blot analysis was conducted using antibodies against μ-calpain and caspase-12. β-actin was used as internal control; (**D**) Cells were pretreated with 5 µM BAPTA/AM for 1 h and then treated with or without 20 µM KSK05104 for 24 h. Cells were co-stained with PI and FITC-conjugated Annexin V, and the translocation of PS was detected via flow cytometry; (**E**) Cells pretreated with 5 µM BAPTA/AM for 1 h and then treated with or without 20 µM KSK05104 for 24 h. Expression of PARP-1 and μ-calpain were examined via Western blot analysis. β-actin was used as internal control. Data presented as means ± S.D. of the results of three independent experiments. ** *p* < 0.01 *** *p* < 0.001 vs. non-treated control group, ^##^ *p* < 0.01, ^###^ *p* < 0.001 vs. KSK05104-treated control group.

ER is an organelle that is involved in protein folding and modification and acts as the main Ca^2+^-storage organelle [28]. ER stress can be caused by disturbances in ER functions due to the accumulation of unfolded proteins and alterations in [Ca^2+^]_i_ homeostasis which is related to cell death. [Ca^2+^]_i_ induces apoptosis in cancer cells by activating Ca^2+^-dependent protein kinases and phosphatases [29]. As shown in Figure 6A, KSK05104 induced ER stress involving activation of PERK and IRE-1α, but there was no effect on ATF6 in HT-29 cells. Because ER is an essential intracellular organelle involved in [Ca^2+^]_i_ homeostasis [15], we determined whether KSK05104-induced ER stress is associated with an increase in [Ca^2+^]_i_. We evaluated the intracellular Fluo-4 fluorescence in terms of its intensity because this is an indicator of the [Ca^2+^]_i_ in HT-29 cells untreated or treated with KSK05104. Following 3 h treatment with 20 µM KSK05104, [Ca^2+^]_i_ became markedly higher than that of control (Figure 6B). In addition, KSK05104 time-dependently induced the activation of μ-calpain and caspase-12 (Figure 6C). In addition, pretreatment with the BAPTA/AM (cell-permeable Ca^2+^ chelator) significantly prevented the KSK05104-induced apoptosis in HT-29 cells (Figure 6D) and pretreatment with BAPTA/AM for 1 h also potently prevented KSK05104-induced activation of PARP-1 and μ-calpain (Figure 6E). These results indicate the involvement of ER stress with KSK05104-induced apoptosis through the elevation of [Ca^2+^]_i_ and its related protease activation in HT-29 cells.

## 3. Discussion

One of the hallmarks of cancer is the escape of tumor cells from cell death, mainly from apoptosis.

The compounds that normally block or suppress cancer cell proliferation by inducing cell cycle arrest and apoptosis possess potential for development into anti-cancer agents [30]. The present results demonstrate that KSK05104 is a potent cytotoxic agent in human colon adenocarcinoma HT-29 and HCT-116 cells, and its cytotoxic effect is associated with the induction of apoptosis. Apoptosis, or programmed cell death, is a mechanism that has been evolutionarily conserved to eliminate unwanted cells that commonly occur during development or in various pathologic and physiological processes [31]. This highly-regulated process consists of cells undergoing inducible non-necrotic cell death. In general, the activation of a cysteine protease family, termed caspases, is important for the apoptosis-triggering mechanisms, and its induction is thought to play a key role in the progression of cancer [32]. A hallmark of apoptosis at the molecular level is the activation of caspase-specific proteases that conduct cell death via cleavage of multiple protein substrates [33]. Caspases can be divided into initiators (caspases-2, -8, -9, and -10) and effectors (caspases-3, -6, and -7) according to their relative position within a caspase cascade [34]. The results of this study showed KSK05104 time-dependently activated caspases-8, -9, and -3, and increased the cleavage of the PARP-1 substrate. Pretreatment with z-VAD-fmk partially inhibited KSK05104-induced apoptosis, indicating that KSK05104 induces caspase-dependent and caspase-independent (AIF-dependent) apoptosis in HT-29 cells.

The Bcl-2 family of proteins controls a critical step toward apoptosis by regulating the permeabilization of the mitochondrial outer membrane [35]. The Bcl-2 family is divided into a pro-apoptotic class that includes Bak, Bax, and Bid and an anti-apoptotic class that includes Bcl-2, Bcl-xL, and Bcl-w [36]. Bax and Bak change their conformation when activated by BH3 domain-only proteins in the family and permeabilize the MOM, while the anti-apoptotic members inhibit permeabilization [37]. In the present study, HT-29 cells were treated with KSK05104, resulting in a decreased expression of Bcl-2 and an increase in the expression of Bak. In mitochondria, the mitochondrial permeability transition, matrix swelling, and outer membrane rupture causes a release in the mitochondrial Cyt *c* [38]. Once in the cytosol, Cyt *c* binds to Apaf-1 in a dATP/ATP dependent manner, which triggers the oligomerization of Apaf-1/Cyt *c* in complexes that activate caspase-9 [39]. The dissipation of *ΔΨ_m_* was significant by 2 h after KSK05104 treatment in HT-29 cells. Released cytosolic Cyt *c* activates caspase-9 by binding to Apaf-1, which leads to caspase-9 activation with subsequent activation of the downstream effector caspases.

A particular mitochondria-dependent step involving outer membrane permeabilization is associated with most pro-apoptotic stimuli, and this process is controlled by pro- and anti-apoptotic members of the Bcl-2 family, which lead to cytosolic release of mitochondrial intermembrane space proteins including AIF, Endo G, and Cyt *c* to trigger either caspase activation or caspase-independent death pathways [40]. Among such mitochondrial proteins, during apoptosis, AIF can induce large DNA fragmentation (~50 kbp), presumably in a caspase-dependent manner, and such fragmentation could be a prerequisite of nucleosomal cleavage by EndoG or other nucleases [41]. In this study, we investigated how mitochondrial AIF, Endo G, and Cyt *c* release involving *ΔΨ_m_* were critical for KSK05104-mediated apoptosis. We confirmed that treatment of KSK05104 increased expression of AIF, Endo G and release of cytochrome c.

Cancer cell treatment with chemotherapeutic agents induces a sustained increase in intracellular Ca^2+^ concentrations resulting from the depletion of ER Ca^2+^ stores [42]. Cellular Ca^2+^ has been strongly implicated in inducing apoptosis and regulation the apoptotic signaling pathways. Cellular Ca^2+^ homeostatic mechanisms include Ca^2+^ entry from the extracellular space through voltage-insensitive and voltage dependent Ca^2+^ channels, Ca^2+^ release from the ER stores, and cytosolic Ca^2+^ buffering, which terminates the Ca^2+^ signal [43]. KSK05104 seems to be selectively associated with an increase in [Ca^2+^]_i_, because the apoptotic activity of KSK05104 was blocked by pretreatment with BAPTA/AM, but not with EGTA (Ca^2+^ influx blocker from extracellular space) (data not shown). Prior studies of ER stress-induced apoptosis in tumor cells suggest the existence of two distinct induction mechanisms. One involves the accumulation of unfolded or misfolded proteins, and the other involves alterations in [Ca^2+^]_i_ [44]. The ER is the primary site for protein synthesis, folding, and trafficking. Under a variety of stressful conditions, the accumulation of unfolded or misfolded proteins in the ER causes the onset of ER stress [13]. In mammalian cells, the main unfolded protein response (UPR) signaling is initiated by three main ER-localized protein sensors: IRE-1α, protein kinase-like ER kinase (PERK), and activating transcription factor 6 (ATF-6) [45]. High level or prolonged activation of the UPR signaling is related to cell death. Thus, the degree and duration of the UPR evaluated by sensors of ER stress (IRE-1 α, PERK and ATF6) seems to be critical for cell death or survival [46]. Without stress, each can be maintained in an inactive state through association with Grp78 (Bip) [47]. The accumulation of unfolded proteins in the ER lumen or the depletion of Ca^2+^ from the ER lumen leads to ER stress responses known as UPR. [Ca^2+^]_i_ incrementally activates μ-calpain which is Ca^2+^-dependent apoptotic protease followed by caspase-12 activation [42,48]. The present study demonstrates that KSK05104 induces activation of ER stress including PERK and IRE-1α, resulting in elevated [Ca^2+^]_i_ levels and μ-calpain and caspase-12 activation.

In conclusion, our results demonstrate that a novel rhodanine derivative KSK05014 induces apoptosis in human colon adenocarcinoma HT-29 cells through both mitochondria dysfunction and ER stress. During KSK05014-induced apoptosis, Bcl-2 family protein modulation leads to a loss of *ΔΨ_m_*, resulting in a release of Cyt *c*, AIF, and Endo G. In addition, KSK05104-induced apoptosis is mediated by the ER stress, such as activation of PERK and IRE-1α, leading to [Ca^2+^]_i_ increase, which subsequently triggers μ-calpain and caspase-12 activation. These results support the further exploration of KSK05014 as a possible chemotherapeutic agent to treat colon cancer.

## 4. Materials and Methods

### 4.1. Chemicals and Reagents

KSK05104 (Figure 1A) was synthesized, and its structural identity was spectroscopically determined. The NMR results are described below. (5-[4-(4-Fluorophenoxy)phenyl]methylene-3-{4-[3-(4-methylpiperazin-1-yl)propoxy]phenyl}-2-thioxo-4-thiazolidinone dihydrochloride): ^1^H NMR (400 MHz, DMSO-*d*_6_) 2.20–2.21 (m, 2H), 2.81 (bs, 3H), 3.34–3.78 (m, 10H), 4.13 (t, *J* = 6.0 Hz, 2H), 7.08–7.12 (m, 4H), 7.18–7.23 (m, 2H), 7.27–7.33 (m, 4H), 7.70 (d, *J* = 9.0 Hz, 2H),7.80 (s, 1H), 11.76 (bs, 2H); ^13^C NMR (100 MHz, DMSO-*d*_6_) 23.8, 42.5, 48.7, 50.1, 53.6, 65.6, 115.4, 117.3, 117.5, 118.4, 121.9, 122.5, 122.6, 128.2, 128.3, 130.4, 132.5, 133.4, 151.4, 158.2, 159.2, 160.1, 160.6, 167.5, 194.5. Materials related to cell culture were obtained from Life Technologies Inc. (Grand Island, NY, USA) and Fluo-4/AM was obtained from Molecular Probes, Inc. (Eugene, OR, USA) Primary antibodies were purchased from Santa Cruz Biotechnology, Inc. (Santa Cruz, CA, USA) and Cell Signaling Technology (Danvers, MA, USA). A broad caspase inhibitor, z-VAD-fmk was purchased from Calbiochem (San Diego, CA, USA) and extraction kit for mitochondria was purchased from Active Motif. All of the other reagents were purchased from Sigma Chemical co. (St. Louis, MO, USA).

### 4.2. Cell Culture

Human colon cancer cell lines HT-29 and HCT-116, human lung epithelial cell line L132 and human colon fibroblast cell line CCD-18Co were obtained from the Korean cell line bank (KCLB, Seoul, Korea) and immortalized ovarian surface epithelial cell line IOSE-80PC was kindly provided by Dr. Auersperg (University of British Columbia, Vancouver, BC, Canada) and A. Godwin (Fox Chase Cancer Center, Philadelphia, PA, USA). Cells were cultured in RPMI 1640 supplemented with 10% heat-inactivated fetal bovine serum (FBS), penicillin (100 units/mL) and streptomycin (100 µg/mL). The cells were cultured at 37 °C in an atmosphere of 5% CO_2_ in the presence or absence of KSK05104.

### 4.3. MTT Assay

To detect cancer cell viability, an MTT assay was performed. Briefly, colon cancer cells (HT-29, HCT-116) and normal cells (IOSE-80PC) were seeded in each well containing 100,000 cells/mL in 100 µL of the medium supplemented with 10% FBS in a 96 well plate. After 24 h, various concentrations of KSK05104 were added. After 48 h, 20 µL of MTT (5 mg/mL stock solution in phosphate buffered saline (PBS)) was added and the plates were incubated for an additional 2 h. The medium was discarded and the formazan blue, which was formed in the cells, was dissolved with dimethyl sulfoxide (DMSO). The optical density was measured at 540 nm using microplate readers (Molecular Devices, San Jose, CA, USA).

### 4.4. Annexin V and PI Double Staining by Flow Cytometry

Assay for apoptosis, annexin V and PI double staining were performed to detect the phosphatidylserine exposure on the exterior surface of the plasma membrane as we previously described [49]. Cells were suspended with 100 µL of binding buffer (10 mM 4-(2-hydroxyethyl)-1-piperazineethanesulfonic acid (HEPES)/NaOH, 140 mM NaCl, 2.5 mM CaCl_2_, pH 7.4) and stained with 5 µL of FITC-conjugated annexin V and 5 µL of PI (50 µg/mL). The mixture was incubated for 15 min at room temperature in a dark place and analyzed by fluorescence-activated cell sorting (FACS) cater-plus flow cytometry (Becton Dickinson Co, Heidelberg, Germany).

### 4.5. Determination of the ΔΨm

To determine the change in the *ΔΨm*, flow cytometry was carried out via DiOC_6_ staining, which uses a mitochondria-specific voltage-dependent dye. After treatment with KSK05104, HT-29 cells were incubated with 40 nM DiOC_6_ for 30 min. The cells were harvested by centrifugation at 1000× *g* for 5 min and washed with ice-cold PBS twice. DiOC_6_ fluorescence was measured using fluorescence-activated cell sorting (FACS) cater-plus Flow cytometry (Beckman Coulter, Brea, CA, USA) with excitation and emission wavelengths of 488 and 530 nm, respectively.

### 4.6. Western Blot Analysis

The extraction of proteins from KSK05104-treated cells and their concentration were prepared and determined as previously reported [49]. KSK05104-treated cells were harvested and washed twice with ice-cold PBS. Cell pellets were resuspended in hypotonic buffer (10 mM HEPES, pH 7.9, 1.5 mM MgCl_2_, 10 mM KCl, 0.2 mM phenylmethylsulfonyl fluoride (PMSF), 0.5 mM dithiothreitol (DTT), 10 μg/mL aprotinin) and incubated on ice for 15 min. Cells were then lysed by adding 0.1% Nonidet P-40 and vortexed vigorously for 10 s. Nuclear fractions were pelleted by centrifugation at 12,000× *g* for 1 min at 4 °C and resuspended in high salt buffer (20 mM HEPES, pH 7.9, 25% glycerol, 400 mM KCl, 1.5 mM MgCl_2_, 0.2 mM EDTA, 0.5 mM DTT, 1 mM NaF and 1 mM sodium orthovanadate). For total cell protein extracts, cell pellets were lysed in ice-cold cell lysis buffer (50 mM HEPES, pH 7.0, 250 mM NaCl, 5 mM EDTA, 0.1% Triton X-100, 0.5 mM DTT, 5 mM NaF, 0.5 mM Na orthovanadate, 0.1 mM PMSF and protease inhibitor cocktail) for 20 min on ice. Cell debris was removed by microcentrifugation (10,000× *g*, 5 min), followed by quick freezing of the supernatants. Protein concentrations were determined using Bio-Rad protein assay reagent, according to the manufacturer’s instruction. Cellular proteins (10–40 µg) from treated or untreated cell extracts was separated on 8–15% SDS-PAGE onto a polyvinylidene difluoride (PVDF), which was incubated for 1 h with blocking solution at 4 °C, and then with primary antibody overnight. Blots were then washed four times with Tween 20/Tris-buffered saline (TBS/T), incubated with horseradish peroxidase-conjugated secondary antibody for 1 h at room temperature, rewashed three times with TBS/T and then developed by enhanced chemiluminescence (Amersham Life Science, London, UK).

### 4.7. Preparation of Cytosolic and Mitochondrial Fractionations

KSK05104 treated cells were washed with ice cold PBS and were extracted using a mitochondrial fractionation kit. The cells were collected by centrifugation (600× *g*, 5 min, 4 °C), washed twice with ice-cold PBS, and then centrifuged again (600× *g*, 5 min, 4 °C). The obtained cell pellet was then resuspended in ice-cold cytosolic buffer for 15 min on ice. The cells were homogenized with a glass dounce and a B-type pestle (80 strokes), homogenates were spun at 10,000× *g* for 20 min at 4 °C, and the supernatant (cytosolic fraction) was removed whilst avoiding the pellet. The resulting pellet (mitochondrial fraction) was re-suspended in a completed mitochondria buffer. Each fraction was then frozen in aliquots at −70 °C until required.

### 4.8. Determination of [Ca^2+^]_i_

[Ca^2+^]_i_ were measured using Ca^2+^-indicator dye Fluo-4/AM (cell membrane permeable fluorescent dye) assay kit (Invitrogen, Carlsbad, CA, USA), according to the manufacturer’s instructions.

### 4.9. Statistical Analysis

The data presented are the means ± S.D. of the results from three independent experiments with similar patterns. * *p* < 0.05, ** *p* < 0.01, *** *p* < 0.001 vs. non-treated control group, *^#^ p* < 0.05, *^##^ p* < 0.01, ^###^ *p* < 0.001 vs. KSK05104-treated control group; significance of difference between treated groups by Student’s *t*-test.

## Figures and Tables

**Table 1:** The cytotoxic activity of KSK05104 assessed by MTT assay on cell growth in vitro.

Cell Line	Origin	IC_50_ (μM)
HT-29	Human colorectal adenocarcinoma	15.93 ± 2.41
HCT-116	Human colorectal carcinoma	31.96 ± 1.56
CCD-18Co	Human colon fibroblast	88.79 ± 2.91
L132	Human lung epithelial cell	90.06 ± 3.55
IOSE-80PC	Immortalized ovarian surface epithelial cell	45.8 ± 3.69
